# Photoinduced Membrane Damage of *E. coli* and *S. aureus* by the Photosensitizer-Antimicrobial Peptide Conjugate Eosin-(KLAKLAK)_2_

**DOI:** 10.1371/journal.pone.0091220

**Published:** 2014-03-07

**Authors:** Gregory A. Johnson, E. Ann Ellis, Hansoo Kim, Nandhini Muthukrishnan, Thomas Snavely, Jean-Philippe Pellois

**Affiliations:** 1 Department of Biochemistry & Biophysics, Texas A&M University, College Station, Texas, United States of America; 2 Microscopy & Imaging Center, Texas A&M University, College Station, Texas, United States of America; MGH, MMS, United States of America

## Abstract

**Background/Objectives:**

Upon irradiation with visible light, the photosensitizer-peptide conjugate eosin-(KLAKLAK)_2_ kills a broad spectrum of bacteria without damaging human cells. Eosin-(KLAKLAK)_2_ therefore represents an interesting lead compound for the treatment of local infection by photodynamic bacterial inactivation. The mechanisms of cellular killing by eosin-(KLAKLAK)_2_, however, remain unclear and this lack of knowledge hampers the development of optimized therapeutic agents. Herein, we investigate the localization of eosin-(KLAKLAK)_2_ in bacteria prior to light treatment and examine the molecular basis for the photodynamic activity of this conjugate.

**Methodology/Principal Findings:**

By employing photooxidation of 3,3-diaminobenzidine (DAB), (scanning) transmission electron microscopy ((S)TEM), and energy dispersive X-ray spectroscopy (EDS) methodologies, eosin-(KLAKLAK)_2_ is visualized at the surface of *E. coli* and *S. aureus* prior to photodynamic irradiation. Subsequent irradiation leads to severe membrane damage. Consistent with these observations, eosin-(KLAKLAK)_2_ binds to liposomes of bacterial lipid composition and causes liposomal leakage upon irradiation. The eosin moiety of the conjugate mediates bacterial killing and lipid bilayer leakage by generating the reactive oxygen species singlet oxygen and superoxide. In contrast, the (KLAKLAK)_2_ moiety targets the photosensitizer to bacterial lipid bilayers. In addition, while (KLAKLAK)_2_ does not disrupt intact liposomes, the peptide accelerates the leakage of photo-oxidized liposomes.

**Conclusions/Significance:**

Together, our results suggest that (KLAKLAK)_2_ promotes the binding of eosin Y to bacteria cell walls and lipid bilayers. Subsequent light irradiation results in membrane damage from the production of both Type I & II photodynamic products. Membrane damage by oxidation is then further aggravated by the (KLAKLAK)_2_ moiety and membrane lysis is accelerated by the peptide. These results therefore establish how photosensitizer and peptide act in synergy to achieve bacterial photo-inactivation. Learning how to exploit and optimize this synergy should lead to the development of future bacterial photoinactivation agents that are effective at low concentrations and at low light doses.

## Introduction

The rising incidence of drug resistant pathogens emphasizes the urgent need for new approaches to antimicrobial killing [1]–[4]. One alternative to traditional antibiotics for topical microbial killing is photodynamic inactivation (PDI), a therapeutic strategy that combines photosensitizers (PS) and light. In this approach, PS are compounds that produce reactive oxygen species (ROS) upon irradiation [5]. These ROS can in turn cause cell death by oxidizing biomolecules such as proteins, nucleic acids, and lipids [5]–[7]. A limitation of PDI consists in the fact that light does not penetrate tissues deeply. PDI is therefore not adequate for the treatment of systemic infections. On the other hand, PDI has been successfully applied to the treatment of acne [8]–[11], oral disinfection [12], peptic, skin, and diabetic foot ulcers [13]–[15], and blood decontamination [16]–[18]. PDI also kills antibiotic resistant strains as effectively as their antibiotic sensitive counterparts [19]–[21], and repeated sub-lethal PDI treatments have failed to produce resistant strains [22]. PDI therefore represents a possible long-term approach for the treatment of local infections. Additionally, applications of PDI to infections of the skin/soft tissues and surgical sites may prove to be particularly valuable when considering that these infections account for ∼7–10% of hospitalized patient infections [23] and 20–31% [24], [25] of healthcare-associated infections, respectively. PDI could play an important role in these contexts to prevent, or reduce the likelihood of, subsequent systemic infections after passage of organisms from the initial infection sites into the bloodstream [26].

A challenge in PDI consists of designing PS that have broad-spectrum activity while also maintaining low phototoxicity towards human cells. PS are often hydrophobic and generally have a significant affinity for biological membranes [27], [28]. Hydrophobic PS are typically capable of binding Gram-positive bacteria and photo-killing is often effective. However, these PS are often not able to kill Gram-negative strains, presumably because the LPS-rich cell wall constitutes a relatively impermeable barrier [29]. In addition, hydrophobic PS often lack specificity in targeting bacterial membranes, leading to unintended binding and damage to human cells [30]. In order to promote binding to the negatively charged surface of bacterial membranes, PS have been conjugated to cationic polymers. For instance, PS have been conjugated to poly-lysine (pL) and poly-ethyleneimine (PEI) [31]–[33], and certain positively charged peptides such as cell-penetrating peptides (CPPs) [34]–[36]. These cationic polymers improve the activity of PS towards Gram-negative strains significantly [36]. However, the phototoxicity of such polymer-PS conjugates towards human cells remains problematic as human cells also have a high propensity to bind and internalize these species [37]–[40].

Recently, the amphipathic antimicrobial peptide (AMP) (KLAKLAK)_2_ conjugated to the photosensitizer eosin Y was designed as a novel PDI agent [41]. This design was guided by the notion that eosin Y, a rather hydrophilic PS, would not significantly associate with membranes on its own. On the other hand, AMPs are known to associate with bacteria more than with human cells. A hypothesis was therefore that (KLAKLAK)_2_ might target eosin Y to bacteria efficiently while avoiding association with human cells. Indeed, efficient binding of eosin-(KLAKLAK)_2_ to both Gram negative *E. coli* and Gram positive *S. aureus* was observed under conditions when eosin Y itself does not associate with bacteria. Consequently, efficient photokilling of bacteria (Gram negative *E. coli*, *A. baumannii*, and *Ps. aeruginosa*, and Gram positive *S. aureus* and *S. epidermidis*) was achieved with eosin-(KLAKLAK)_2_ upon light irradiation while similar treatments with eosin Y did not cause cell killing. In contrast, eosin-(KLAKLAK)_2_ did not significantly associate with human cells (i.e. plasma membrane binding and endocytic uptake are limited) and the photokilling of human cells was minimal at the concentrations for which more than 99.99% bacterial killing is achieved (e.g. 1 µM eosin-(KLAKLAK)_2_ for 5-log reduction of 10^8^ CFU/mL *E. coli* or *S. aureus* cultures). However, while these results are promising, a 10-fold increase in the concentration of eosin-(KLAKLAK)_2_ caused significant photohemolysis of human red blood cells (RBCs) and phototoxicity to certain cell lines. The photokilling specificity achieved with eosin-(KLAKLAK)_2_ is therefore not ideal and optimizing the activity of this compound further would presumably be valuable for *in vivo* applications.

To improve the activity of eosin-(KLAKLAK)_2_, a path forward involves understanding its mechanism of action as a basis for future rational design. In this report, our goal was thus to gain a molecular understanding of how eosin-(KLAKLAK)_2_ causes bacterial photoinactivation. In particular, because AMPs such as (KLAKLAK)_2_ are often thought to interact with bacterial lipid bilayers, we test the hypothesis that eosin-(KLAKLAK)_2_ destroys bacterial membranes. By exploiting DAB photooxidation and STEM/EDS techniques for electron microscopy, we are able to gain unprecedented visualization of a PS-AMP in its cellular context. Additionally, bacteria killing and *in vitro* liposome assays suggest plausible molecular targets, ROS mechanisms, and molecular properties of eosin-(KLAKLAK)_2_ during bacterial photoinactivation.

## Materials and Methods

### Materials

Fmoc amino acids and HBTU were purchased from Novabiochem, while solvents and chemicals were purchase from Sigma. One exception was 5(6)-carboxy eosin Y, which was purchased from Marker Gene Technologies. For liposome preparation, 1-stearoyl-2-oleoyl-sn-glycero-3-phosphocholine (PC), cholesterol (Chol), choline sphingomyelin (SM), dioleoyl-phosphatidyl ethanolamine (PE), L-α-Phosphatidyl-DL-Glycerol (PG), and cardiolipin (CA) were purchased from Avanti Lipids.

### Solid Phase Peptide Synthesis

The antimicrobial peptide H_2_N-KLAKLAKKLAKLAK-NH_2_, or “(KLAKLAK)_2_” was synthesized by Fmoc chemistry, as described previously. [41] The conjugate eosin-(KLAKLAK)_2_ was obtained by coupling of 5,6-carboxy-eosin Y to the N-terminus of the peptide. The compound was purified by reversed-phase C18 HPLC and characterized by MALDI-TOF, as described previously [41].

### Light Source for Photodynamic Experiments

Irradiation was achieved using a homemade setup with a 600 W halogen lamp (Utilitech #0320777). [41] To prevent overheating of the lamp, the glass face was removed and air-cooled during operation. The lamp was suspended over a homemade water filter to remove heat from infrared wavelengths by continuous exchange of the water supply. A stir plate was placed underneath the water filter to hold samples during illumination. Samples were placed in wells of a 96-well plate with micro stir bars and a lid. A 5×7 inch green filter (Edmund Optics cat. no. NT46–624, 470–550 nm FWHM) was placed on top of the lid for excitation of eosin. A single pane of 1/16 inch diffusing glass was placed on top of the green filter to provide an even distribution of light intensity. Experiments detecting the ^1^O_2_ production from Rose Bengal via reaction with RNO (p-nitrosodimethylaniline) demonstrated that this setup provides even distribution of light across all 96 wells (data not shown). Samples were stirred at 200 rpm and set at a distance of 20 cm from the light source. Irradiance at this distance through all filters was 131 mW/cm^2^ (∼236 J/cm^2^ for a 30 min exposure) as determined with a Newport 840-C optical power meter. For experiments with Ce6, a similar red filter was used (Edmund Optics cat. no. NT46–622, ∼625 nm cut-on filter).

### Bacterial Strains

*Escherichia coli* BL21 DE3 was obtained from Agilent, and *Staphylococcus aureus* subsp. *aureus* (ATCC 29213) was purchased from the American Type Culture Collection. *E. coli* and *S. aureus* were grown in Luria-Bertani broth (LB). Glycerol stocks were established for each strain and used to streak agar plates. Colonies from plates were used to inoculate overnight cultures that were grown aerobically at 37°C. Fresh cultures were inoculated the next day in a 1∶1000 dilution of overnight culture and used for experiments after growth to mid log phase (O.D._600_ ∼0.4–0.6, corresponding to ∼10^9^ CFU/ml).

### Photooxidation, Fixation, and DAB Polymerization in Bacteria Samples

Samples of *E. coli* or *S. aureus* were prepared in the same manner used previously for phototoxicity experiments [41]. Cultures were grown overnight in LB broth and fresh subcultures were prepared in the morning. After growth to O.D.600 ∼0.6, the cells were pelleted and resuspended in phosphate buffer (100 mM NaCl, 10 mM Na_2_HPO_4_, pH 7.4), and this wash procedure repeated once more. The stock suspension was diluted to an O.D. which gave approximately 10^8^ CFU/ml for each strain. Eosin-(KLAKLAK)_2_ (22 µl of 10 µM), or H_2_O as a blank, was added to wells of a 96 well plate before addition of 200 µl of bacteria suspension in phosphate buffer (10^8^ CFU/ml). Samples were prepared in duplicate and kept in the dark for 2 min or illuminated under the halogen lamp assembly mentioned above for 2 or 5 min. Acrolein (100 µl of 2% solution) was then added to samples and incubated at room temperature for 20 min to fix the bacteria and any bound eosin-(KLAKLAK)_2_. To remove unbound eosin-(KLAKLAK)_2_, the samples were transferred to microcentrifuge tubes and pelleted in a small bench top centrifuge for 5 min. The supernatant was removed and samples were washed twice with 100 µl of cold 0.1 M cacodylate buffer. The pellets were then resuspended in the same buffer supplemented with 0.1 M glycine to react with any remaining acrolein in solution, and allowed to stand for 20 min before addition of 100 µl of diaminobenzidine (DAB) buffer (1 mg/ml DAB in cacodylate buffer). These suspensions were transferred to a 96 well plate for 15 min illumination to polymerize DAB specifically in the locations where the peptide was fixed, followed by an additional 100 µl of DAB buffer and 15 min of illumination. Samples were then transferred back to microcentrifuge tubes and washed twice with cacodylate buffer, followed by suspension in cacodylate buffer containing 1% (wt/vol) osmium tetroxide.

### Electron Microscopy Sample Preparation and Imaging

After suspension of cells in osmium tetroxide, samples were dehydrated with 10% steps of methanol to (10%–100%), infiltrated overnight, and embedded in Quetol 651-Spurr epoxy resin [42] and polymerized overnight. Thin sections (200–250 nm) were cut with a Microstar diamond knife, (Huntsville, TX) using an AO Ultracut ultramicrotome picked up on grids which were carbon stabilized with approximately 10 nm of carbon using a Cressington 308 evaporative coater. Bright field images were obtained using a JEOL 1200 EX TEM (tungsten filament electron gun, 120 keV accelerating voltage). For each sample, ∼25–50 cells were imaged, and representative images were chosen for each population. Elemental analysis was performed on an FEI TECNAI F20 Super Twin (scanning) transmission electron microscope ((S)TEM) fitted with a Schottky field emission gun, a high angle annular dark field (HAADF) detector, and an EDAX instrument ultrathin window energy dispersive X-ray spectroscopy (EDS) detector. The combination of STEM and EDS allows direct imaging of a nanoscale area and *in situ* identification of component elements. Darkfield images were taken by the HAADF detector in STEM mode. Approximately 40 cells were imaged in this manner before selecting duplicate representative cells for EDS analysis. Representative EDS data from a single sample was chosen for publication. An EDS spectrum at each spot in the area of interest was collected at a 200 kV accelerating voltage and a ∼15° tilting angle with a stationary electron probe in STEM mode to see component elements. Elemental line profiles were then acquired after choosing a proper energy window for each element-specific energy transition.

### In vitro Detection of Singlet Oxygen and Superoxide Production

Detection of singlet oxygen from eosin Y or eosin-(KLAKLAK)_2_ was achieved by irradiation in the presence of imidazole and RNO (p-nitrosodimethylaniline) [43]. Production of singlet oxygen from eosin Y leads to reaction with imidazole to form a peroxide intermediate, which subsequently reacts with RNO to cause bleaching of RNO absorbance. A total reaction volume of 200 µl was obtained by addition of 20 µl each of 10X solutions for RNO, imidazole, quencher (or H_2_O blank), PS or PS-AMP (or H_2_O blank), and 120 µl phosphate buffer (10 mM phosphate, pH 7.4, 100 mM NaCl). Final concentrations were 50 µM RNO, 8 mM imidazole, 100 mM sodium azide, and eosin Y or eosin-(KLAKLAK)_2_ at 1 or 10 µM. Illumination was carried out in the same manner as bacterial killing experiments to ensure relevant results. Bleaching of RNO was detected at 450 nm using a Glomax Multi+Plate reader.

Detection of superoxide was achieved by excitation of eosin Y and eosin-(KLAKLAK)_2_ in the presence of NADH and NBT (nitro blue tetrazolium). A total reaction volume was obtained with 10X stock solutions in the manner mentioned above for the RNO assay. Final concentrations for eosin Y or eosin-(KLAKLAK)_2_ were 1 or 10 µM, 10 mM NADH, and 80 µM NBT. Illumination was carried out in the same manner as bacterial killing experiments. Reduction of NBT resulting in the production of a formazan was detected by absorbance at 600 nm using a plate reader. Since the RNO and NBT reactions proceed by oxidation and reduction, respectively, there is no cross talk between the assays [33], [44].

### Bacterial Killing Experiments with ROS Quenchers

Bacterial killing experiments were carried out in the same manner as described previously [41]. Peptide and quencher solutions were placed in wells of a 96 well plate, composed of 11 µl of 20X quencher with and without 11 µl of 20X eosin-(KLAKLAK)_2_ or H_2_O blanks where appropriate, before addition of 200 µl of bacteria culture (10^8^ CFU/ml). Crocetin was used from a 100X stock in DMSO, requiring only 2.2 µl of stock in the same total volume of 222 µl. Samples were allowed to incubate for approximately 3–5 min before irradiation to allow for peptide binding, and micro stir bars (2×2 mm, Cowie via Fisher) were added for continued aeration during irradiation. The lipid to peptide (L/P) ratio under these conditions is 1∶1 when the peptide or PS concentration is approximately 3 µM (these calculations assume 25×10^6^ lipids per bacteria). Samples were irradiated with the same setup described above with a 30 min exposure for each sample.

After samples were illuminated for 30 min, 30 µl of each sample was added to 270 µl of phosphate buffer in a separate 96-well plate. Further 10-fold serial dilutions of the samples were made in phosphate buffer to give samples ranging from 10^1^–10^5^ in dilution factor. From each dilution, 50 µl was removed and spread on an agar plate, then incubated overnight at 37°C. Colonies were counted the next morning to determine the remaining CFU/mL. Plates without peptide treatment were included as a negative control for sample comparison to determine percent survival.

### Liposome Preparation

Large unilamellar vesicles (LUVs) of two compositions were prepared to represent the lipids and net surface charge of human [45], [46] and bacterial (Gram negative *E. coli* [47] and Gram positive *S. aureus* [48]) membranes. The human (Hum) LUVs were a 50/30/20 ratio of PC/Chol/SM. The bacterial (Bac) composition was 75/20/5 of PE/PG/CA. The outer membrane of *S. aureus* presents a significantly greater negative charge than this composition due to higher PG content (>75%), no PE, and little lysyl PG (3%) [48]. These Bac LUVs therefore present a lower threshold of negative charge, and likewise, charge-based attraction for the cationic (KLAKLAK)_2_. Since the ROS produced by photooxidation mechanisms target carbon-carbon double bonds [49], the double bond content should also be noted in the design. The PC, Chol, and SM component of Hum LUVs each contain one double bond. The PE and PG lipids of Bac LUVs also have a single unsaturation. CA has four double bonds per molecule but represents only 5% of the total LUV lipids, reflective of bacterial composition [47], [48]. Overall, for a LUV sample of 100 µmole total lipid, there are therefore 100 µmole of unsaturated sites for Hum LUVs and 115 µmole for Bac LUVs.

Stock lipids in chloroform were mixed in a glass vial for the required molar ratios and the solvent evaporated under a nitrogen stream. Lipid mixtures were then placed in a vacuum desiccator for a minimum of 2 hrs before addition of phosphate buffer (10 mM phosphate, pH 7.4, 100 mM NaCl; 60 mM calcein included as needed for leakage assays). The lipids were then put through ten freeze-thaw cycles between liquid nitrogen and a water bath at 42°C to create multi-lamellar vesicles (MLVs). These MLVs were extruded twenty one times using an Avanti extruder with a 100 nm polycarbonate membrane. For LUVs without calcein, these were transferred to a glass vial for storage under nitrogen at 4°C. LUVs with calcein required separation of external dye by size exclusion chromatography using a Sephadex G-50 column in phosphate buffer. The calcein-loaded LUV preparations for both lipid compositions used for this manuscript were stable for approximately two-four weeks when kept at 4°C. The stability varies with lipid composition, and should be monitored in each case. We monitored stability over time by measuring the increase in fluorescence of calcein-loaded LUVs after addition of 0.1% Triton X-100 (final concentration), using a 200 µl sample of 200 µM total lipid. Samples were placed in a 96 well plate and fluorescence determined with a Promega Glomax Multi microplate reader. Ten-fold dilutions were made where needed to ensure that no self-quenching remained in the detergent samples, allowing for a linear comparison between samples.

### Leakage Assays

For leakage experiments, stock solutions of calcein-loaded LUVs were diluted as needed in phosphate buffer to obtain working solutions of 200 µM total lipid. Wells of a 96 well plate were first filled with 11 µl of 20X quencher or H_2_O blank, followed by 11 µl of 20X eosin-(KLAKLAK)_2_ or H_2_O blank. A volume of 200 µl of the 200 µM LUV working solution was then added to each well. This mixture provides a 1X concentration of quencher and eosin-(KLAKLAK)_2_, with 90% of the total lipid concentration in the final solution. Samples were irradiated using the light source described above, and calcein release was monitored by fluorescence with a plate reader (Ex 490, Em 510–570). Readings of all samples were taken before irradiation for intensities at 0 min.

Leakage experiments testing the specific role of the (KLAKLAK)_2_ peptide in the lysis of bacterial (Bac) LUVs were performed in a similar manner as described above. For experiments with chlorin e6 (Ce6), Bac LUVs were kept in the dark or irradiated for 10 min with either a water blank or Ce6 (10 µM) alone. After the first dark or irradiated step, a water blank or (KLAKLAK)_2_ (1, or 10 µM) was added to each sample for 20 min before reading the fluorescence again to assess any additional leakage caused by (KLAKLAK)_2_.

### Fluorescence Anisotropy Measurements

Peptide and photosensitizer binding to LUVs can be described by K, the apparent molar partition coefficient [50], [51]. The total lipid concentration [L] during measurements was significantly higher than peptide concentration bound to liposomes [P]_b_, therefore binding can be defined as:
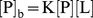
(1)where [P] is the free molar peptide concentration in solution. Since the peptide or photosensitizer could potentially cross the membrane, [L] is the total lipid concentration in solution. Substitution of [P] in equation (1) with [P]_tot_-[P]_b_, followed by algebraic rearrangement leads to an expression of fraction bound ([P]_b_/[P]_tot_):



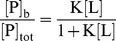
(2)To obtain values of K, binding of peptide or photosensitizer was determined by titration with model LUVs of bacterial or mammalian lipid composition, and the fluorescence anisotropy recorded for different total lipid concentrations. Fluorescence anisotropy measurements were recorded in L-format using a SLM-8000C fluorometer (SLM Instruments, Bath, UK) with the Phoenix package (ISS, Champaign, IL) and Vinci v.1.6 PC software (ISS). Samples were excited at 525 nm and emission detected through a 560 cut-on filter for eosin Y and eosin-(KLAKLAK)_2_, or a 590 cut-on filter for Ce6, using 0.5 mm slits on each side. To remove scattering background, blank titrations were performed with LUVs alone. The parallel and perpendicular emission intensities (vertical-vertical and vertical-horizontal polarizer positions, respectively) of the blanks were subtracted from those of the samples at each lipid concentration, before calculation of the steady state anisotropy (r) using the equation:
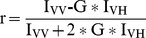
(3)where the subscript pairs denote the vertical (V) or horizontal (H) orientation of the excitation and emission polarizers, respectively, for the detected intensities (I). The instrument-specific parameter G = I_HV_/I_HH_ corrects for detector sensitivity to vertically and horizontally polarized light [52]. Titrations were repeated at least twice to obtain average r values for each point. To correct for changes in quantum yield after binding to membranes and also account for the contribution of anisotropy from free and bound forms, the fraction bound f_B_ was calculated using the equation:
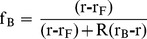
(4)where rF and rB are the anisotropy values for fully free and bound (saturated) states, r is the anisotropy value at each titration step, and R = IB/IF is the ratio of total intensities from the bound (saturated) and free states, respectively. Total intensity was calculated as shown in the denominator of equation 3, and all values were corrected for dilution resulting from titration. Binding curves were plotted and fit to a single site-specific binding model with Hill slope using GraphPad Prism v.6 software. The reciprocal of K, the dissociation constant Kd, was obtained from the curve fits for comparison of binding affinities. Kd values describe the total molar concentration of lipid in solution required to achieve 50% fraction bound for the fluorophore or peptide.

### Statistical Analysis

Data were processed using Microsoft Excel. Experiments were performed in triplicate unless otherwise noted. The average values of three or more replicate experiments were computed with error bars representing the standard deviations. In order to curve fit the binding anisotropy data, average anisotropy and standard deviation values were transferred into GraphPad Prism. This program computes the value and error of Kd based on the curve fit of the averaged data and the corresponding error values.

## Results

### Eosin-(KLAKLAK)_2_ Localizes to the Outer Surface of *E. Coli* and *S. Aureus* in the Dark, and Subsequent Light Excitation causes Membrane Disruption

To investigate the mechanism of bacterial photoinactivation by eosin-(KLAKLAK)_2_, we first sought to determine where the compound localizes in bacteria. To achieve this aim, the DAB photooxidation methodology developed for TEM was adapted herein [53], [54]. In this approach, ROS-generating species can be localized with high resolution by detecting the ROS-induced polymerization of 3,3-diaminobenzidine (DAB). The DAB polymer is osmiophilic and increased osmium staining at the site of polymer formation is visualized as dark contrast by electron microscopy [53], [55]. Oxidizing ROS do not typically diffuse far away from their site of generation and DAB polymerization is therefore restricted to where the ROS-generating moiety is [53], [55]. Eosin Y has been previously used to induce DAB polymerization [53]. We therefore expected that eosin-(KLAKLAK)_2_ could also lead to DAB polymerization and that a dark contrast visualized by EM would indicate where eosin-(KLAKLAK)_2_ localizes in bacteria.

**Figure 1:**
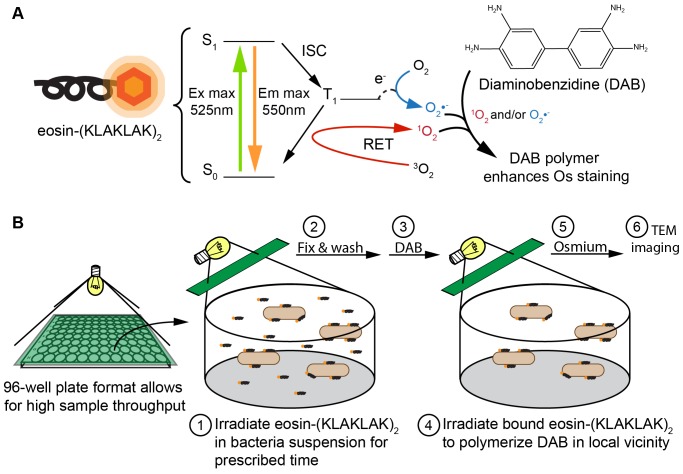
Experimental design of DAB photo-oxidation and visualization by TEM. (A) Light excitation of eosin-(KLAKLAK)_2_ results in production of singlet oxygen and superoxide, which can polymerize DAB to provide an enhanced staining of osmium at the location of eosin-(KLAKLAK)_2_. (B) Light irradiation has two purposes in this experiment, 1) to excite eosin-(KLAKLAK)_2_ for photodynamic activity (step 1), then following fixation of samples, 2) to polymerize DAB at the location of the PS-AMP conjugate (step 4).

**Figure 2:**
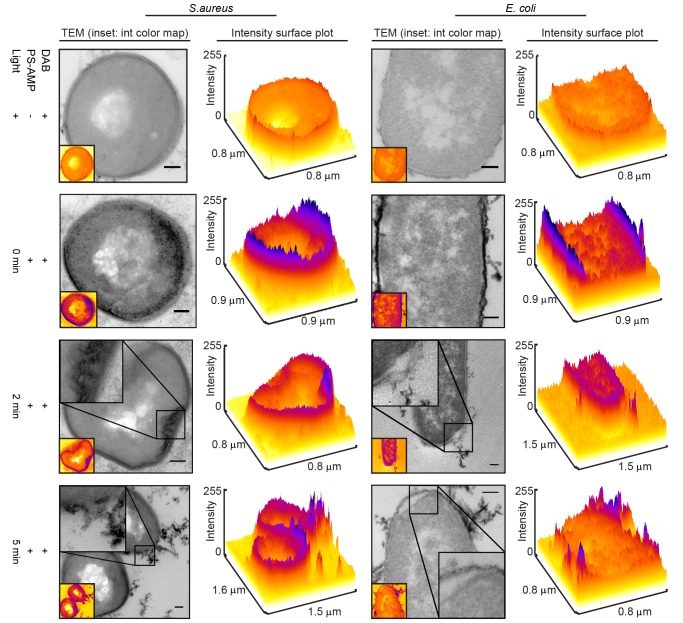
Localization of eosin-(KLAKLAK)_2_ in *S. aureus* and *E. coli* samples determined by DAB photooxidation. Control samples without eosin-(KLAKLAK)_2_ are shown in the top row. For remaining samples, eosin-(KLAKLAK)_2_ was incubated with cells, then irradiated for 0, 2, or 5 min (2^nd^, 3^rd^, and 4^th^ rows, respectively) before fixation with acrolein, anchoring the bound eosin-(KLAKLAK)_2_ in place. Cells were then washed and a second illumination was performed in the presence of DAB (1 mg/ml), producing an osmiophilic polymer for enhanced contrast by TEM at the site of eosin-(KLAKLAK)_2_. To facilitate sample comparison, intensity surface plots were rendered from the 8-bit images shown, using a FIRE LUT and the surface plot tool in ImageJ. All scale bars are 0.1 µm.

Figure 1 depicts the adapted DAB polymerization protocol followed in our experiments [53], [54], [56]. Eosin-(KLAKLAK)_2_ was mixed with *E. coli* or *S. aureus* and the samples were fixed with acrolein. Control samples without eosin-(KLAKLAK)_2_ were also prepared. The fixed samples were treated with DAB buffer and illuminated with the filtered halogen lamp (Figure S1) to induce DAB polymerization. The resulting DAB staining and cell morphologies are shown by TEM images in Figure 2, along with their corresponding intensity surface plot profiles for simplified comparison. Samples treated with eosin-(KLAKLAK)_2_ show a very dark contrast at the cell walls while cells not exposed to the peptide do not (these samples are still treated with DAB and irradiated).

To visualize the photo-damage caused by eosin-(KLAKLAK)_2_ during bacterial photoinactivation, samples of eosin-(KLAKLAK)_2_ and bacteria were illuminated for 2 or 5 min prior to fixation. Under these conditions, approximately 50% and 90% cell death is obtained for both *E. coli* and *S. aureus*, as previously reported [41]. A longer irradiation time of 30 min results in a 5 log reduction (99.999%) of the same cultures. Our rationale was that under conditions of shorter irradiation time, the early stages of photo-damage that lead to cell death would be observed as opposed to photo-damage events that might take place well after cells are dead. As shown in the third and fourth rows of Figure 2, light irradiation of eosin-(KLAKLAK)_2_ prior to fixation, results in membrane damage and lysis of the cell wall. At 2 min irradiation, deformation of the cell wall of *S. aureus* can be observed. Under similar condition, *E. coli* cells display rupture of the outer and inner membranes. At 5 min irradiation, large structures with dark contrast form on the surface of both strains and membrane damage is more severe. It is also interesting to note that DAB contrast diminishes in certain samples (e.g. *E. coli* at 2 min). This is expected however as the eosin Y moiety of eosin-(KLAKLAK)_2_ will partially photobleach during the irradiation step required for cell killing and thereby have a reduced ability to cause DAB polymerization in subsequent steps.

**Figure 3:**
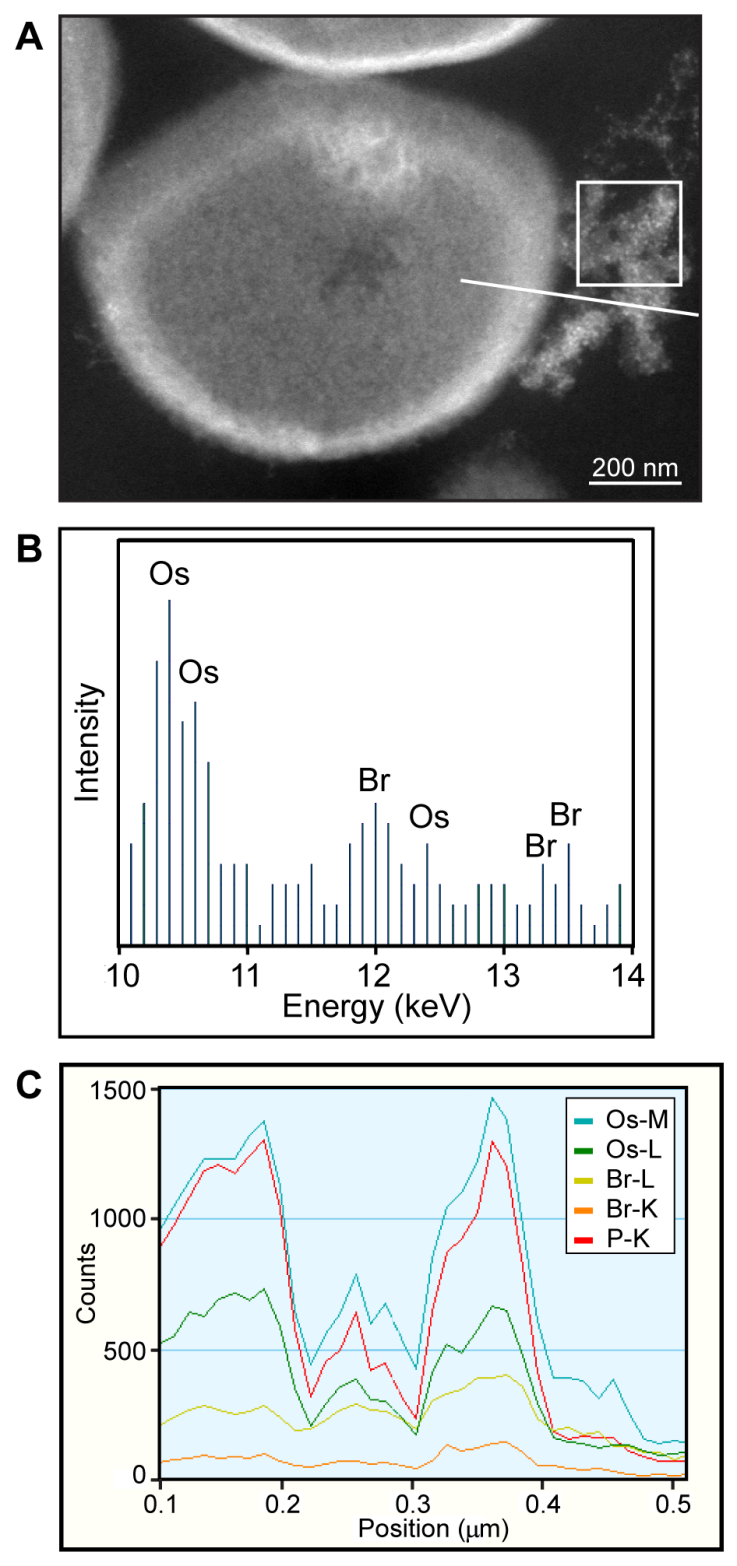
Bromine atoms from eosin-(KLAKLAK)_2_ serve as a marker for detection by STEM-EDS in bacteria samples. (A) STEM dark field image of *S. aureus* treated with eosin-(KLAKLAK)_2_ and light for 2 min. (B) Elemental analysis by EDS for the square area indicated in (A), showing the distinct presence of Br from eosin-(KLAKLAK)_2_ (the transitions of C, O, P, and other elements are predominant at lower energy levels and thus not seen here). (C) EDS element profiles of the line scan depicted in (A), showing the coincident intensities of Os, Br, and P elements at the interior, cell wall, and extracellular material, with more than 250 counts for Br at the membrane and extracellular regions.

In order to confirm that the enhanced osmium staining at the cell surface of bacteria was in fact the result of the presence of eosin-(KLAKLAK)_2_, scanning TEM (STEM) with energy dispersive X-ray spectroscopy (EDS) was used. STEM-EDS is a methodology that can be used to analyze the distribution of select atoms in biological samples [57], [58]. Eosin Y contains four bromine atoms per molecule. In contrast, bromine is a rare element in most bacterial species and bromine is not detected by STEM-EDS in *E. coli* or *S. aureus* [59]. Bromine can therefore act as a specific marker for the location of eosin-(KLAKLAK)_2_ [60]. In Figure 3a an image of an *S. aureus* cell treated with eosin-(KLAKLAK)_2_ and light for 2 min is shown. A white box depicts the location of an area scan at what appears to be adjacent membrane debris in the media, with the resulting elemental profile shown in Figure 3b. Br atoms are detected, indicating the presence of eosin-(KLAKLAK)_2_ in this extracellular debris. A white line is also shown in Figure 3a which depicts the path of a line scan, sampling the cell and extracellular debris for STEM-EDS analysis. The intensity of bromine content is depicted along the path of the line scan (from left to right) in Figure 3c. The bromine intensity is greatest at the cell membrane and at the location of the adjacent debris. Additionally, bromine intensity also correlates with that of osmium.

### Eosin-(KLAKLAK)_2_ causes Leakage of Liposomes of Bacterial, but not Mammalian, Lipid Composition in the Presence of Light

**Figure 4:**
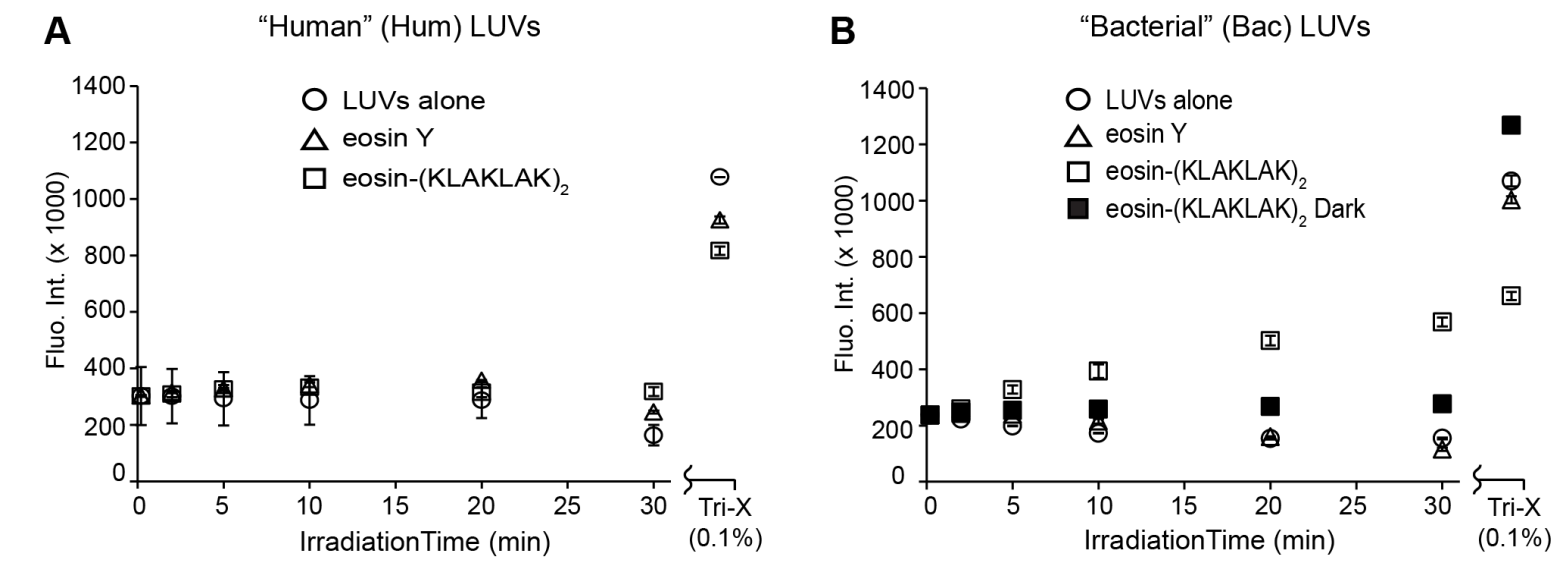
Eosin-(KLAKLAK)_2_ lyses LUVs of bacterial lipid composition, but not of mammalian composition. Eosin-(KLAKLAK)_2_ (10 µM) was mixed with LUVs (200 µM) of (A) “Human” (Hum) or (B) “Bacterial” (Bac) lipid composition, each containing a self-quenching concentration of calcein (60 mM). Samples were irradiated for the times indicated and leakage was detected as an increase in fluorescence intensity after release of calcein and subsequent unquenching. Average values are shown for triplicate experiments with error bars representing the standard deviation.

Eosin-(KLAKLAK)_2_ binds to and photo-destroys the cell walls of both Gram positive and negative strains. ROS characterization showed that both singlet oxygen (^1^O_2_) and superoxide (O_2_^•−^) might mediate these effects. This is based on dependence for oxygen and inhibition of killing by ROS quenchers (Figure S2). Additionally, both ^1^O_2_ and O_2_^•−^ are generated by eosin-(KLAKLAK)_2_ (Figure S3). However, since eosin Y and eosin-(KLAKLAK)_2_ show similar ROS production at the low concentrations (1 µM) used for bacterial killing, the activity of eosin-(KLAKLAK)_2_ cannot be fully explained by ROS generation (eosin also binds to *E. coli* at high concentration but does not induce cell killing [41]). In order to gain further insights in the photo-killing mediated by eosin-(KLAKLAK)_2_, it seemed reasonable to test how the conjugate interacts with a common component of the two cell walls, namely, the lipid bilayer. To test whether eosin-(KLAKLAK)_2_ damages lipid bilayers upon irradiation, leakage assays using calcein loaded LUVs were first performed (Figure 4). Disruption of LUVs in this system results in the release of calcein with subsequent un-quenching and an increase in fluorescence. Calcein-loaded LUVs (100 nm diameter, 200 µM total lipid) with or without eosin-(KLAKLAK)_2_ present in solution (10 µM), were irradiated for 30 min under the same conditions as bacterial killing assays. Values for 100% lysis were determined by addition of 0.1% Triton X-100 to LUVs to release the remaining calcein. LUVs with a lipid composition representative of bacterial lipid bilayers were used along with LUVs characteristic of human plasma membranes as a control. These LUVs in particular differ in charge as the lipids of bacterial (Bac) LUVs are negatively charged while the lipids of mammalian (Hum) LUVs are neutral.

Figure 4a shows that light alone or the combination of light and Eosin Y (10 µM) does not cause leakage for either type of LUV. In contrast, irradiation of eosin-(KLAKLAK)_2_ leads to early and continued leakage from Bac LUVs (Figure 4b), while no such leakage was observed without irradiation. Interestingly, no leakage was observed with Hum LUVs. After the addition of Triton X-100, it is apparent that the total fluorescence of LUVs treated with eosin-(KLAKLAK)_2_ is significantly diminished compared to LUVs alone or with eosin Y, indicating significant bleaching of calcein caused by eosin-(KLAKLAK)_2_ during irradiation. This suggests that the apparent fluorescence of calcein (and thus apparent leakage) throughout the irradiation process is actually underestimated for eosin-(KLAKLAK)_2_ in this assay. The apparent leakage during irradiation nonetheless provides a lower limit for the extent of leakage achieved.

**Figure 5:**
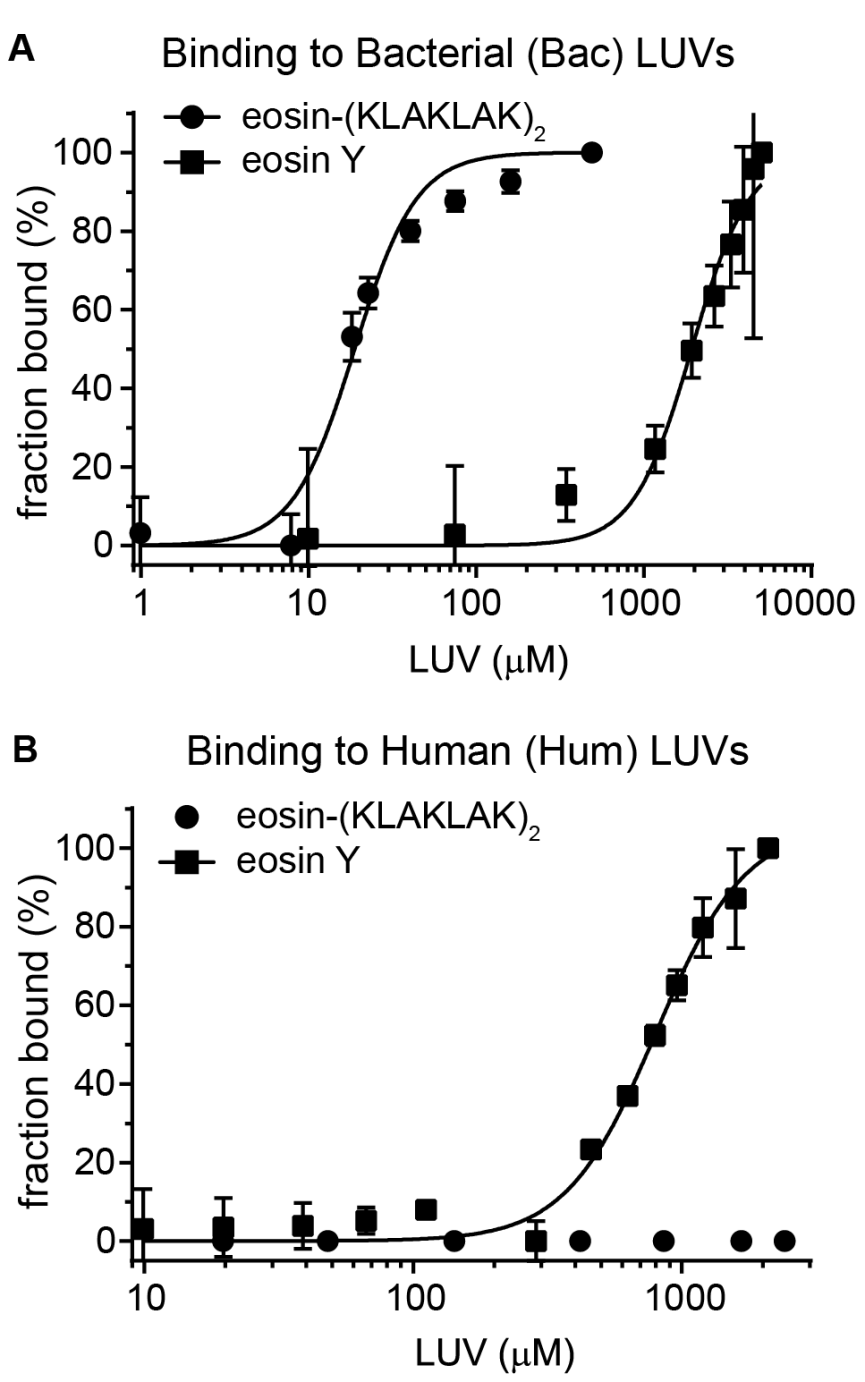
Eosin-(KLAKLAK)_2_ binds to LUVs of bacterial, but not human lipid composition. Model liposomes of bacterial (A) or human (B) lipid composition were titrated into a solution of 1 µM eosin Y or eosin-(KLAKLAK)_2_ to detect changes in anisotropy. The resulting values were used to calculate the fraction bound and the data were fit to a single-site binding model with Hill slope. Eosin-(KLAKLAK)_2_ showed no change in anisotropy after addition of Hum LUVs.

In order to establish why eosin Y and eosin-(KLAKLAK)_2_ differ in activity and why eosin-(KLAKLAK)_2_ disrupts Bac LUVs but not Hum LUVs, steady state fluorescence anisotropy was used to test the binding of these compounds to LUVs (Figure 5). Addition of Bac LUVs, but not Hum LUVs, to eosin-(KLAKLAK)_2_ resulted in a significant increase in anisotropy. The data were best fit by a single-site binding model with Hill slope, displaying an apparent cooperativity as seen previously for lysine-containing peptides binding to acidic liposomes [61], [62]. As shown in Table 1, eosin-(KLAKLAK)_2_ (net charge +6 at pH 7.4) associates with negatively-charged Bac LUVs (Kd = 18.8+/−0.948 µM). but not with neutral Hum LUVs. In contrast, Eosin Y alone (net charge −2 at pH 7.4 [63]) binds to neutral Hum LUVs (Kd = 800+/−49.7 µM), and associates only weakly with negatively charged Bac LUVs (Kd = 1,931+/−183.4 µM).

**Table 1:** Binding parameters derived after curve fitting to anisotropy binding data.

	Dissociation constant, Kd (µM)	
Ligand	Bacterial LUVs	Human LUVs
eosin-(KLAKLAK)_2_	18.8±0.948	Binding not observed
eosin Y	1,931±183.4	800±49.7

### The AMP Component of the Eosin-(KLAKLAK)_2_ Conjugate Actively Participates in Membrane Lysis

**Figure 6:**
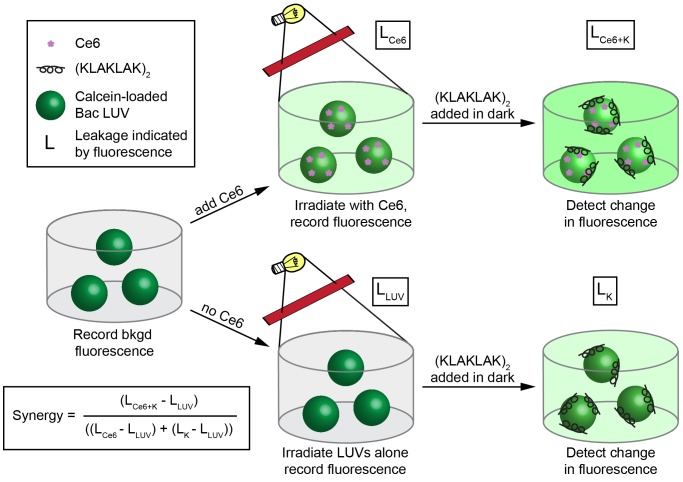
Experimental design to determine the capacity of (KLAKLAK)_2_ for membrane lysis. Bac LUVs containing self-quenching concentrations of calcein were first treated with light in the presence or absence of the PS Ce6, in order to mimic the lipid photooxidation that occurs from irradiation of the eosin-(KLAKLAK)_2_ conjugate. The fluorescence intensity was recorded before and after irradiation to determine any leakage caused by light alone (L_LUV_) or by irradiation of Ce6 (L_Ce6_). Continual fluorescence readings after this point demonstrated that leakage did not persist after the light was turned off (data not shown). A water blank, 1 and 10 µM (KLAKLAK)_2_ (final concentration) was then added in the dark and after 20 min, the fluorescence was read again to determine any additional leakage caused by (KLAKLAK)_2_. The synergy of (KLAKLAK)_2_ with Ce6 was calculated with the equation shown.

AMPs such as (KLAKLAK)_2_ are known to induce liposomal leakage on their own at high P:L ratios. Moreover, it has been recently shown that the ROS-induced oxidation of lipids can enhance the lytic activity of cell-penetrating peptides [40]. We therefore hypothesized that the peptide moiety of eosin-(KLAKLAK)_2_ might promote similar effects and accelerate the leakage of LUV containing oxidized lipids. To test this hypothesis, the experimental protocol presented in Figure 6 was followed. In this scheme, liposomes were first pre-oxidized with the PS chlorin e6 (Ce6) (Figure S1) and subsequently treated with (KLAKLAK)_2_. Unlike eosin Y, Ce6 binds Bac LUVs (Figure S4) and cause leakage upon irradiation. Like eosin Y, Ce6 generates both singlet oxygen and superoxide [33]. Ce6 was therefore used in place of the eosin Y to cause the photo-oxidation of lipids in a manner similar to what is obtained upon irradiation of eosin-(KLAKLAK)_2_.

Leakage from Bac LUVs treated with Ce6 and irradiated for 10 min was first measured by monitoring calcein leakage, as described in Figure 6. After this preliminary step, (KLAKLAK)_2_ (1 or 10 µM) was added to the samples and subsequent LUV leakage was further monitored. In control samples, LUVs were irradiated in the absence of Ce6 but subsequently treated with (KLAKLAK)_2_. The leakage obtained in irradiated samples treated with both Ce6 and peptide was then compared to that obtained in samples treated with peptide alone. In addition, parallel experiments were performed without irradiation in order to assess the membrane leakage that might simply happen by combining Ce6 and (KLAKLAK)_2_ in the dark.

**Figure 7:**
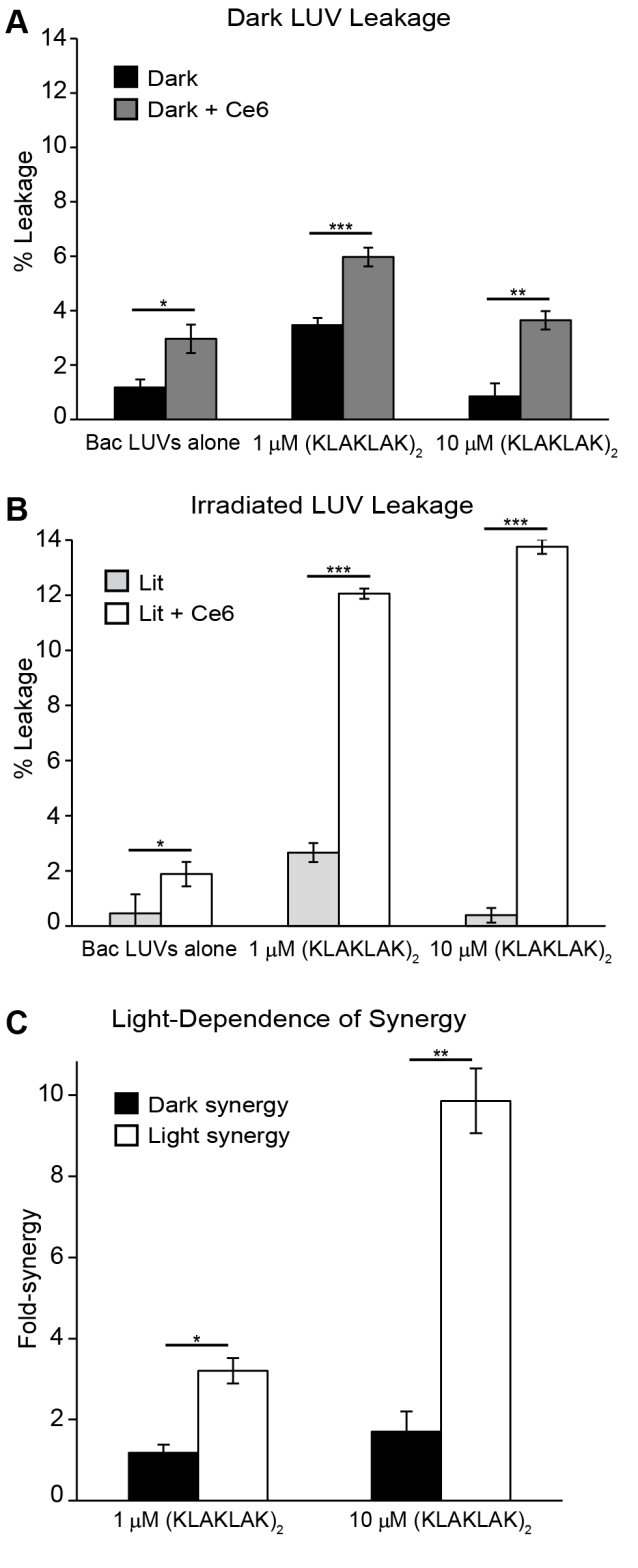
Ce6 and (KLAKLAK)_2_ display synergistic leakage activity towards Bac LUVs. (A) Bac LUVs in the presence or absence of Ce6 were kept in the dark for 10 min before addition of 0, 1, or 10 µM (KLAKLAK)_2_. (B) Same as in (A), but samples were irradiated with light for 10 min before addition of (KLAKLAK)_2_ (two-tailed *t* test, * = p<0.05, ** = <0.01, *** = p<0.001). (C) Synergy of (KLAKLAK)_2_ and Ce6 leakage determined for light and dark conditions using values from (A) and (B).

Figure 7a shows the percent leakage of Bac LUV samples prepared and kept in the dark for 10 min with or without Ce6 (dark gray and black bars, respectively) before subsequent addition of a water blank or (KLAKLAK)_2_ (1 or 10 µM). Ce6 alone showed no significant leakage activity while (KLAKLAK)_2_ led to only 3 and 1% leakage at 1 and 10 µM, respectively. Leakage in the presence of both Ce6 and (KLAKLAK)_2_ was slightly greater, with 8% and 5% leakage obtained at 1 and 10 µM, respectively. Interestingly, leakage was significantly enhanced upon irradiation, as shown in Figure 7b. In particular, irradiation of LUVs incubated with Ce6 alone displayed less than 2% leakage (the irradiation dose was chosen so as to limit lysis by Ce6 alone). Leakage with (KLAKLAK)_2_ alone was observed to be the same as that observed in the dark, as expected for an agent that does not depend on light for its activity. However, samples irradiated with Ce6 and receiving a subsequent addition of (KLAKLAK)_2_ displayed significant enhancements in leakage over those observed for Ce6 or (KLAKLAK)_2_ alone, with 12 and 13% leakage observed at 1 and 10 µM, respectively.

In order to quantify the increased membrane disruption observed upon combining Ce6 and (KLAKLAK)_2,_ synergy was calculated as the ratio L_Ce6+K_/(L_Ce6_+ L_K_), where *L_Ce6_*, *L_K_*, and *L_Ce6+K_*, represent the percent leakage in the presence of Ce6 alone, (KLAKLAK)_2_ alone, and with co-incubation of Ce6 and (KLAKLAK)_2_, respectively. Where synergy exists, leakage obtained by co-incubation of Ce6 and peptide should be greater than the sum of what is obtained with each molecule alone and result in a synergy with a value greater than 1. The results of the calculation for each concentration of (KLAKLAK)_2_ are shown in Figure 7c. Under these conditions, the addition of (KLAKLAK)_2_ results in a synergistic leakage for both dark and light irradiated conditions. This synergy increases with peptide concentration. The synergy observed under irradiated conditions is greater than that observed in the dark for both concentrations, and shows a greater concentration dependent response with light irradiation. Overall, these results indicate that (KLAKLAK)_2_ contributes to increasing the leakage of photo-oxidized liposomes.

## Discussion

In this investigation, we aimed to identify how the conjugate eosin-(KLAKLAK)_2_, a compound containing both an ROS-generator and an antimicrobial peptide, kills bacteria. To begin our investigation, we first aimed to establish how eosin-(KLAKLAK)_2_ associates with bacteria by using electron microscopy (EM) so as to image the distribution of peptide at relatively high resolution. Immunogold staining has been recently used to visualize an AMP by EM [64]. Yet, this approach might not faithfully report on the distribution of a peptide as the relatively large antibodies used for labeling might not be able to reach small peptide targets in the matrix which results from the chemical fixation of samples [53]. To circumvent this problem, we first adapted the DAB photooxidation method to detect the localization of the compound in bacteria. The DAB photooxidation technique has previously been used to determine the localization of large protein complexes [53] or lipophilic dyes [65], but to our knowledge, has never before been used to elucidate information about relatively small peptides. A concern in using this method was that eosin-(KLAKLAK)_2_ might damage cells during DAB polymerization and thus interfere with interpretation. However, in samples not irradiated with light before fixation, the cell morphology appears unaffected, suggesting that the cell is protected from visible damage by the acrolein fixation step that precedes the irradiation required for DAB polymerization. Additionally, control samples without eosin-(KLAKLAK)_2_ showed no contrast, despite also being treated with DAB and irradiated, demonstrating that eosin-(KLAKLAK)_2_ was essential, and that light and DAB alone did not contribute to the contrast. Since DAB photooxidation is a secondary detection method, the presence of Br in the structure of eosin Y was also used to detect eosin-(KLAKLAK)_2_ by STEM-EDS. Notably, the peaks of bromine intensity were found to correlate with those of osmium. This in turn suggest that the regions of dark contrast observed in Figure 2 represent regions where eosin-(KLAKLAK)_2_ is located, as opposed to DAB polymerization occurring at a distant location from eosin-(KLAKLAK)_2_. Overall, these results therefore demonstrated that the DAB photooxidation approach could be successfully applied to this problem. Additionally, while the current study specifically investigates a conjugate with a PS in its native structure, analogous applications of DAB photooxidation and STEM/EDS may also have broad implications for understanding how AMPs and their peptidomimetic counterparts act against a variety of clinically relevant microbes.

DAB photooxidation experiments revealed that the vast majority of eosin-(KLAKLAK)_2_, is localized to the surface of Gram negative *E. coli*, suggesting interaction with the LPS-rich outer membrane. However, similar binding was also observed for Gram positive *S. aureus*, suggesting that components other than LPS might also be capable of interacting with eosin-(KLAKLAK)_2_. Subsequent light irradiation resulted in cell wall damage to both strains. In particular, disruption of the cytoplasmic membrane could be observed in both strains, indicating that lysis of this membrane might be a primary mechanism of cell death. Given that the lipid components of bacterial cell membranes are thought to play a significant role in the activity of both photosensitizers [66], [67] and AMPs [68]–[70], we next tested the binding and leakage activity of eosin-(KLAKLAK)_2_ towards LUVs of bacterial lipid composition. Additionally, since eosin-(KLAKLAK)_2_ displays a selective killing of bacteria over human cells [41], LUVs of mammalian lipid composition were tested as controls. The lipids chosen for Bac LUVs were PE:PG:CA (75∶20: 5), a composition that mimics the negatively charged lipid bilayers of *E.coli* [47] and *S. aureus* [48]. In contrast, the membrane composition chosen for human lipid bilayers was PC:Chol:SM (50∶30: 20), a composition consistent with the outer leaflet of the plasma membrane of human hepatocytes [45], [46] and similar to human red blood cells [71], [72], which show closer to equal levels of PC and SM. While cholesterol might contribute to stabilizing the lipid bilayer, the cholesterol peroxides formed upon reaction with ROS are well-known to be lytic [73]. The Hum LUVs prepared should therefore be susceptible to oxidative damage and lysis. Importantly, unsaturated fatty acids are also known to be oxidized by ROS and their oxidation contributes to lipid bilayer lysis [66], [67]. The number of potential oxidizable unsaturated bonds between Hum and Bac LUVs was therefore chosen to be within the same order of magnitude (∼50% of fatty acid chains contain one unsaturation). These unsaturation levels are also representative of the unsaturation levels present in human membranes (∼50% of fatty acid chains, plus cholesterol) [71] or in the membrane of *E. coli* (50–55%) [74]. It is important to note however that only 2–4% of the fatty acids present in *S. aureus* are monoenoic [75], [76]. Our Bac LUVs are therefore presumably less representative of the complex lipid bilayer of this bacterium. Yet, *S. aureus* also contains unsaturated menaquinones with eight isoprene units, thereby greatly increasing the total amount of oxidizable sites [77]. Admittedly, the propensity for oxidation of each lipid as well as their propensity to induce lipid bilayer disruption might be very different. However, these factors remain largely uncharacterized. With these limitations in mind, our Hum and Bac LUVs should therefore be viewed as simplified membrane models with comparative value.

Eosin-(KLAKLAK)_2_ showed binding to bacterial LUVs, but not to human LUVs. This is consistent with the notion that the positively charged peptide preferentially interacts with negatively charged lipid bilayers rather than zwitterionic bilayers representative of the outer leaflet of human membranes [78]. In addition, irradiation of eosin-(KLAKLAK)_2_ caused leakage of bacterial LUVs but did not affect human LUVs. This is turn validates the notion that the lipid bilayer of bacteria is a potential target of the activity of eosin-(KLAKLAK)_2_. These results also provide a possible explanation for the selectivity observed in light-induced photo-killing. Interestingly, eosin Y alone showed little binding to bacterial LUVs (∼100 fold lower affinity than eosin-(KLAKLAK)_2_) and did not cause leakage upon irradiation. Because Eosin Y and eosin-(KLAKLAK)_2_ were found to generate ROS in similar yields (Figure S2), these results indicate that (KLAKLAK)_2_ increases the photolytic activity of Eosin Y by bringing the PS in close proximity to the lipid bilayer. Additionally, these data suggest that ROS generated in solution by unbound eosin Y or eosin-(KLAKLAK)_2_ do not contribute significantly to leakage.

The LUV binding and leakage results herein support the conclusions of our previous work [41], where eosin-(KLAKLAK)_2_ displayed a strong preference for binding and damaging bacterial cells over mammalian cells. In particular, while irradiation of eosin-(KLAKLAK)_2_ at high concentration (>5 µM) can cause hemolysis, the conjugate does not significantly bind to or lyse red blood cells at concentrations sufficient to kill bacteria (e.g. 1 µM). Moreover, irradiation of eosin-(KLAKLAK)_2_ yielded little to no toxicity toward the human cell lines COLO 316 and HaCaT (up to 10 µM). However, it is interesting to note that the viability of COS-7 treated with 5 and 10 µM eosin-(KLAKLAK)_2_ then irradiated with light was dramatically reduced in comparison to the other cell lines. The decreased viability of COS-7 may suggest a relatively more susceptible lipid composition, or alternatively, the presence of other cellular factors which increase sensitivity to eosin-(KLAKLAK)_2_ photooxidation.

The cell-penetrating peptides TAT and R9 have recently been shown to promote the lysis of oxidized membranes [40], [79]. Although the CPPs alone caused little lysis to RBCs, their addition to RBCs during or after irradiation with rose bengal, enhanced RBC lysis. These peptides thereby displayed a latent membrane disrupting activity towards oxidized membranes. Because AMPs and CPPs possess some structural and functional similarities [80], and because AMPs have an intrinsic lytic activity, we tested the hypothesis that synergy might also take place with (KLAKLAK)_2_. To test for synergy, we examined the leakage of calcein from liposomes of the same bacterial composition used for binding experiments. Liposomes were first irradiated with Ce6 to oxidize the lipid bilayers before addition of (KLAKLAK)_2_. The resulting leakage was compared with that caused by the PS or AMP alone to calculate potential synergy. Interestingly, a synergistic effect was observed when LUVs pre-oxidized by irradiation of Ce6 were then treated with (KLAKLAK)_2_. This result suggests that the PS-AMP conjugate eosin-(KLAKLAK)_2_ might display a similar behavior during photoinactivation of bacteria. For example, one might envision a sequence of events where 1) binding and specificity is first dictated by the AMP, 2) irradiation leads to production of ^1^O_2_ and O_2_^•−^ and thus oxidation of the membrane, 3) resulting in an increased susceptibility of the membrane to the lytic activity of (KLAKLAK)_2_. Additionally, membrane disruption by (KLAKLAK)_2_ could expose new targets to subsequent photodynamic damage, continuing this potential cycle until targets are exhausted or the PS-AMP itself is rendered ineffective by its own ROS production or cellular degradation.

Together, our results establish that eosin-(KLAKLAK)_2_ associates with the cell wall of both Gram positive and Gram negative bacteria. Upon irradiation, eosin-(KLAKLAK)_2_ is capable of destroying membrane components. In particular, disruption of lipid bilayers is observed by EM, and the photo-destruction of liposomes of bacterial lipid composition can be achieved *in vitro*. While eosin Y produces ROS, the peptide moiety (KLAKLAK)_2_ appears to drive the association of the PS with membrane lipids. Interestingly, (KLAKLAK)_2_ is also capable of accelerating lipid bilayer lysis once photo-oxidation of lipids is initiated, presenting a remarkable duplicity to the nature of (KLAKLAK)_2_ interaction with membranes.

Our data suggests that one of the roles played by (KLAKLAK)_2_ is a targeting agent for membrane binding, which is expected since AMPs are well known to interact with and disrupt bacterial lipid bilayers and model lipid systems. Accumulation of AMPs at the membrane surface is typically electrostatically driven in bacteria and liposome models, and can cause membrane disruption by differing mechanisms upon reaching a critical peptide to lipid (P:L) ratio [68]. MD simulations with micelle models also predicted that a short amphipathic helical AMP could deform negatively charged SDS micelles without affecting neutral micelle structure [81]. The P:L ratios for the bacterial killing experiments herein are 1–2 orders of magnitude lower than required to achieve killing in the dark [41], suggesting that under these conditions, (KLAKLAK)_2_ initially serves only as a targeting agent. Furthermore, eosin-(KLAKLAK)_2_ does not cause leakage to Bac LUVs in the dark. Based on the binding affinity of eosin-(KLAKLAK)_2_ measured with Bac LUVs, one molecule is bound for every 22 lipids under the conditions used for LUV leakage experiments. If we assume that the membrane is not crossed by the peptide and consider only the outer leaflet, this corresponds to 11 lipids for every bound peptide. Using the dimensions of lipids (65 Å^2^, ∼9 Å diameter) [82] and a 14a.a. helix (21 Å long, ∼18 Å wide if lysine side chains extend in opposing directions for a 180° polar face [83]), one can estimate that the peptide alone would occupy an area close to that of 5 interspaced lipids. With eosin attached, the structure is extended by ∼10 Å in length and width, so that the eosin-(KLAKLAK)_2_ structure would occupy the area of ∼7 lipids. These numbers suggest that eosin-(KLAKLAK)_2_ occupied approximately two-thirds of the membrane surface. However, despite this high density, no leakage is observed in the dark, supporting the idea that the (KLAKLAK)_2_ moiety serves, at least initially, only as a targeting agent and is otherwise inactive before membrane oxidation occurs.

In addition to membrane targeting, our data suggests that (KLAKLAK)_2_ might play another important role by accelerating the disruption of oxidized membranes. Interestingly, oxidized lipids can also display a lytic activity on their own [67]. It would therefore seem that the latent membrane disrupting activity of (KLAKLAK)_2_ is amplified by oxidation of lipids, or that, conversely, the lytic activity of oxidized lipids is amplified by the AMP. The precise molecular details involved in this synergy remain to be characterized. Nonetheless, these findings are important as they lead to new hypotheses on how to increase the activity of PDI agents. Future studies will examine the potential of rationally designed conjugates for therapeutic applications. In addition, it is interesting to note that ROS production and oxidative damage take place in bacteria constitutively [84], [85]. It is therefore interesting to speculate that oxidized lipids present in bacterial membranes might be involved in bacterial cell death observed upon exposure to AMPs in general (in the dark).

## Supporting Information

Figure S1 **Spectral properties of lamp, filters, and reagents.** (A) Halogen lamp emission spectra through water (heat sink) and color filters (shorter wavelengths limited by detector). (B) Normalized absorbance of eosin-(KLAKLAK)_2_ and eosin Y (left axis), and transmittance of the green filter alone (right axis) used for their excitation. (C) Normalized absorbance of Ce6 (left axis), and transmittance of the applied red filter alone (right axis).
(TIF)

Figure S2 **Role of ROS in eosin-(KLAKLAK)_2_ (“PS-AMP”)-mediated killing of** ***S. aureus*** **(A) and** ***E. coli*** **(B).** Samples (10^8^ CFU/ml) were irradiated with light for 30 min. Serial dilutions were made for colony counting and the survival fraction determined by comparison with non-irradiated controls. Samples without the PS-AMP are included to indicate the toxicity of the quenchers alone. A protective effect against eosin-(KLAKLAK)_2_ by the quencher is indicated by a survival fraction that is greater than the control. Where the quencher alone is non-toxic, yet enhances killing in the presence of eosin-(KLAKLAK)_2_, the quencher could be protecting eosin-(KLAKLAK)_2_ from self-bleaching. (N_2_): Partial displacement of O_2_ was achieved by bubbling N_2_ into the re-suspension buffer. (Imidazole, 50 mM): soluble ^1^O_2_ quencher. (Crocetin, 50 µM): membrane-soluble ^1^O_2_ quencher. (Tiron, 10 mM): soluble O_2_^•−^ quencher; also chelates ions, resulting in cell death to *E. coli*. (Mannitol, 50 mM): soluble HO^•^ quencher. Both strains are protected after oxygen displacement, supporting a direct role for O_2_ in the PDI mechanism (^1^O_2_, Type II), emphasized by crocetin protection (and imidazole in the case of *E. coli*). Additionally, the significant protection of *S. aureus* by Tiron also indicates a Type I (O_2_^•−^) PDI mechanism at work. Although eosin Y is known to produce both ^1^O_2_ and O_2_^•−^, their prospective roles in toxicity have not been demonstrated for eosin-(KLAKLAK)_2_, and interestingly, eosin Y alone displays no toxicity.
(TIF)

Figure S3 **Detection of ^1^O_2_ and O_2_^•^** ^−^ **production from eosin-(KLAKLAK)_2_ and eosin Y.** (A) Relative production of ^1^O_2_ from eosin Y and eosin-(KLAKLAK)_2_ detected by oxidation of RNO in the presence of imidazole. Addition of NaN_3_, a quencher of ^1^O_2_, results in a large reduction of the response. (B) Relative production of O_2_^•−^ from eosin Y and eosin-(KLAKLAK)_2_ detected by reduction of NBT to blue formazan in the presence of NADH, and specific quenching of O_2_^•−^ by Tiron.
(TIF)

Figure S4 **Ce6 binds to Bacterial (Bac) LUVs.** Ce6 (1 µM) was titrated with Bac LUVs in triplicate and the anisotropy data was recorded. The fraction bound was calculated and plotted as averages with their standard deviation. The data was fit to a single site binding model with Hill slope in order to compare with eosin-(KLAKLAK)_2_.
(TIF)
