# Supplementary figures and images for: Photoinduced Membrane Damage of E. coli and S. aureus by the Photosensitizer-Antimicrobial Peptide Conjugate Eosin-(KLAKLAK)2
Source: PLoS One. 2014 Mar 7;9(3):e91220. doi: 10.1371/journal.pone.0091220 (PMC3946741; doi:10.1371/journal.pone.0091220)

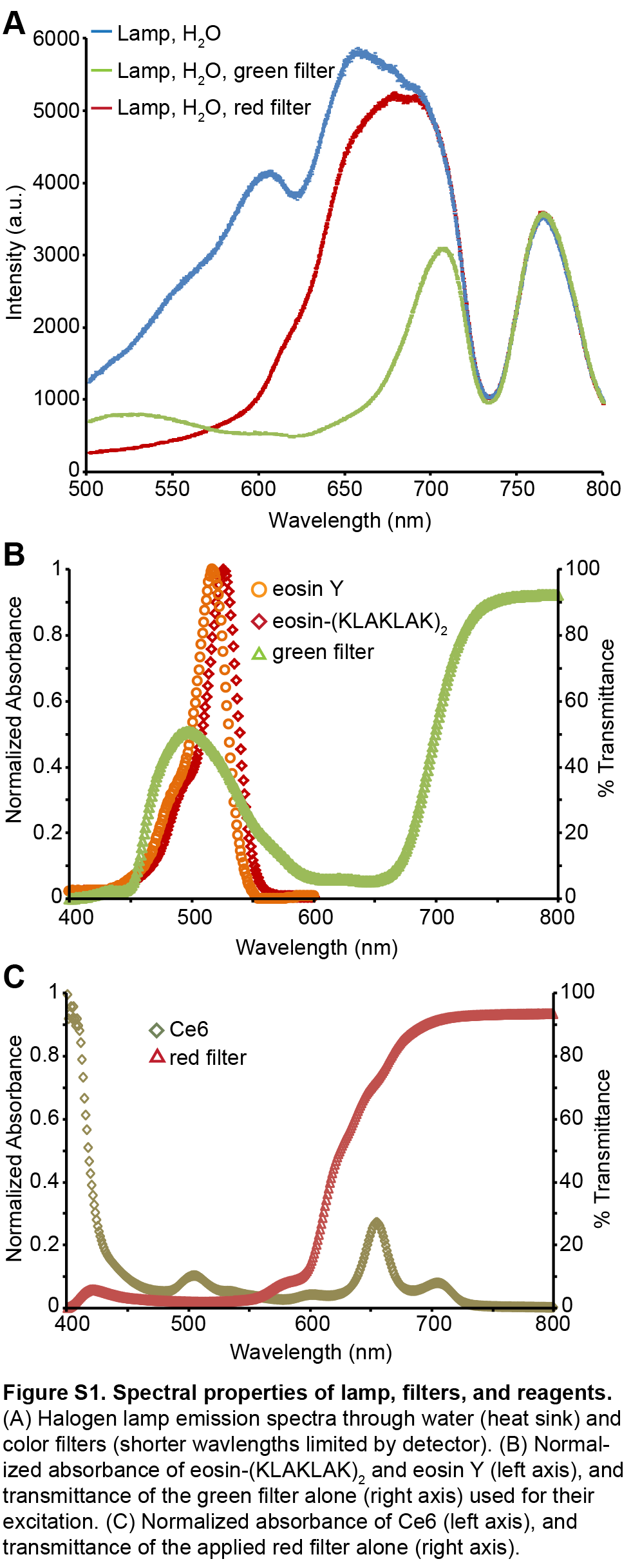

Supplement: Figure S1 — Spectral properties of lamp, filters, and reagents. (A) Halogen lamp emission spectra through water (heat sink) and color filters (shorter wavelengths limited by detector). (B) Normalized absorbance of eosin-(KLAKLAK)2 and eosin Y (left axis), and transmittance of the green filter alone (right axis) used for their excitation. (C) Normalized absorbance of Ce6 (left axis), and transmittance of the applied red filter alone (right axis). (TIF) [file pone.0091220.s001.tif]

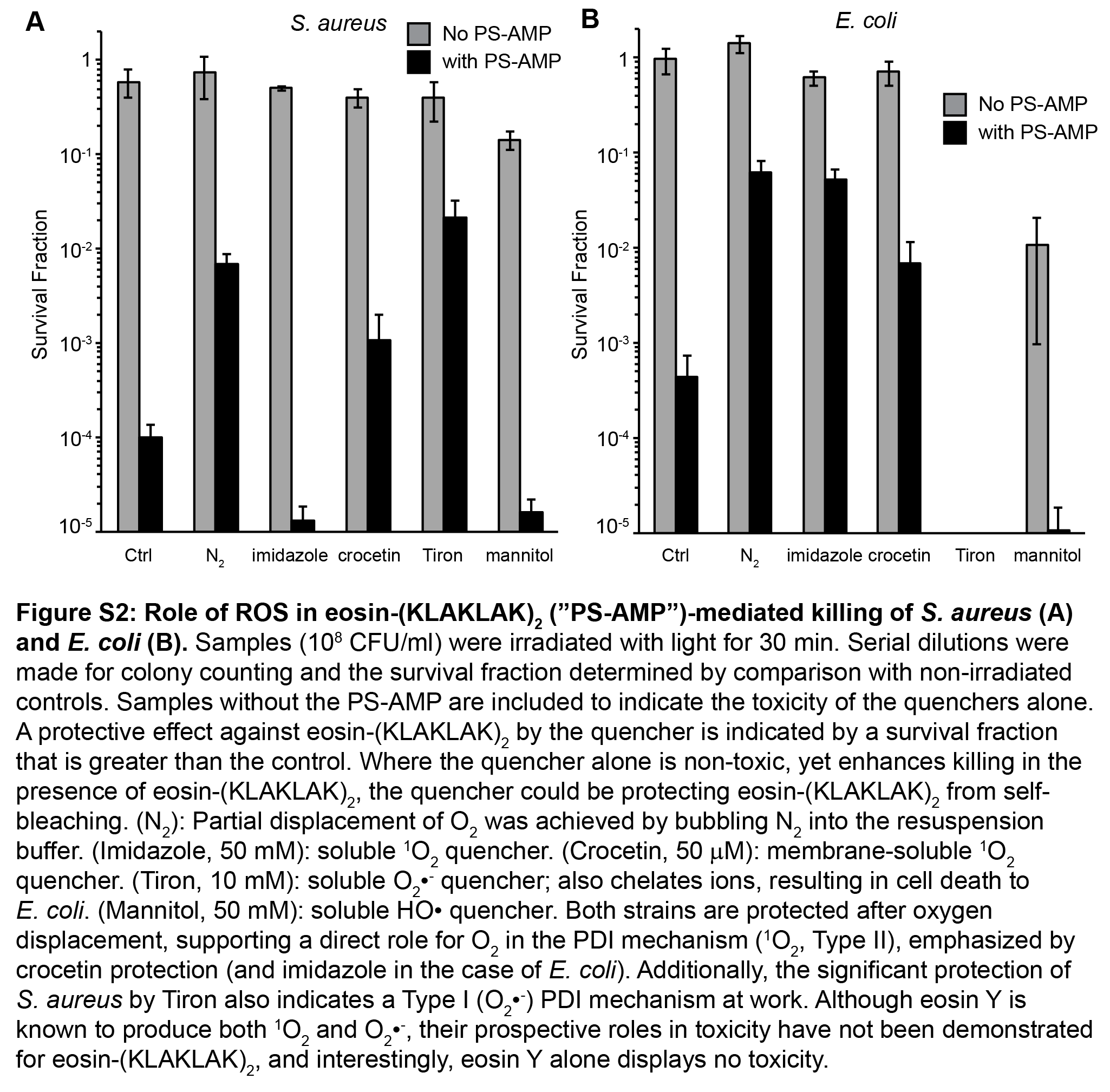

Supplement: Figure S2 — Role of ROS in eosin-(KLAKLAK)2 (“PS-AMP”)-mediated killing of S. aureus (A) and E. coli (B). Samples (108 CFU/ml) were irradiated with light for 30 min. Serial dilutions were made for colony counting and the survival fraction determined by comparison with non-irradiated controls. Samples without the PS-AMP are included to indicate the toxicity of the quenchers alone. A protective effect against eosin-(KLAKLAK)2 by the quencher is indicated by a survival fraction that is greater than the control. Where the quencher alone is non-toxic, yet enhances killing in the presence of eosin-(KLAKLAK)2, the quencher could be protecting eosin-(KLAKLAK)2 from self-bleaching. (N2): Partial displacement of O2 was achieved by bubbling N2 into the re-suspension buffer. (Imidazole, 50 mM): soluble 1O2 quencher. (Crocetin, 50 µM): membrane-soluble 1O2 quencher. (Tiron, 10 mM): soluble O2 •− quencher; also chelates ions, resulting in cell death to E. coli. (Mannitol, 50 mM): soluble HO• quencher. Both strains are protected after oxygen displacement, supporting a direct role for O2 in the PDI mechanism (1O2, Type II), emphasized by crocetin protection (and imidazole in the case of E. coli). Additionally, the significant protection of S. aureus by Tiron also indicates a Type I (O2 •−) PDI mechanism at work. Although eosin Y is known to produce both 1O2 and O2 •−, their prospective roles in toxicity have not been demonstrated for eosin-(KLAKLAK)2, and interestingly, eosin Y alone displays no toxicity. (TIF) [file pone.0091220.s002.tif]

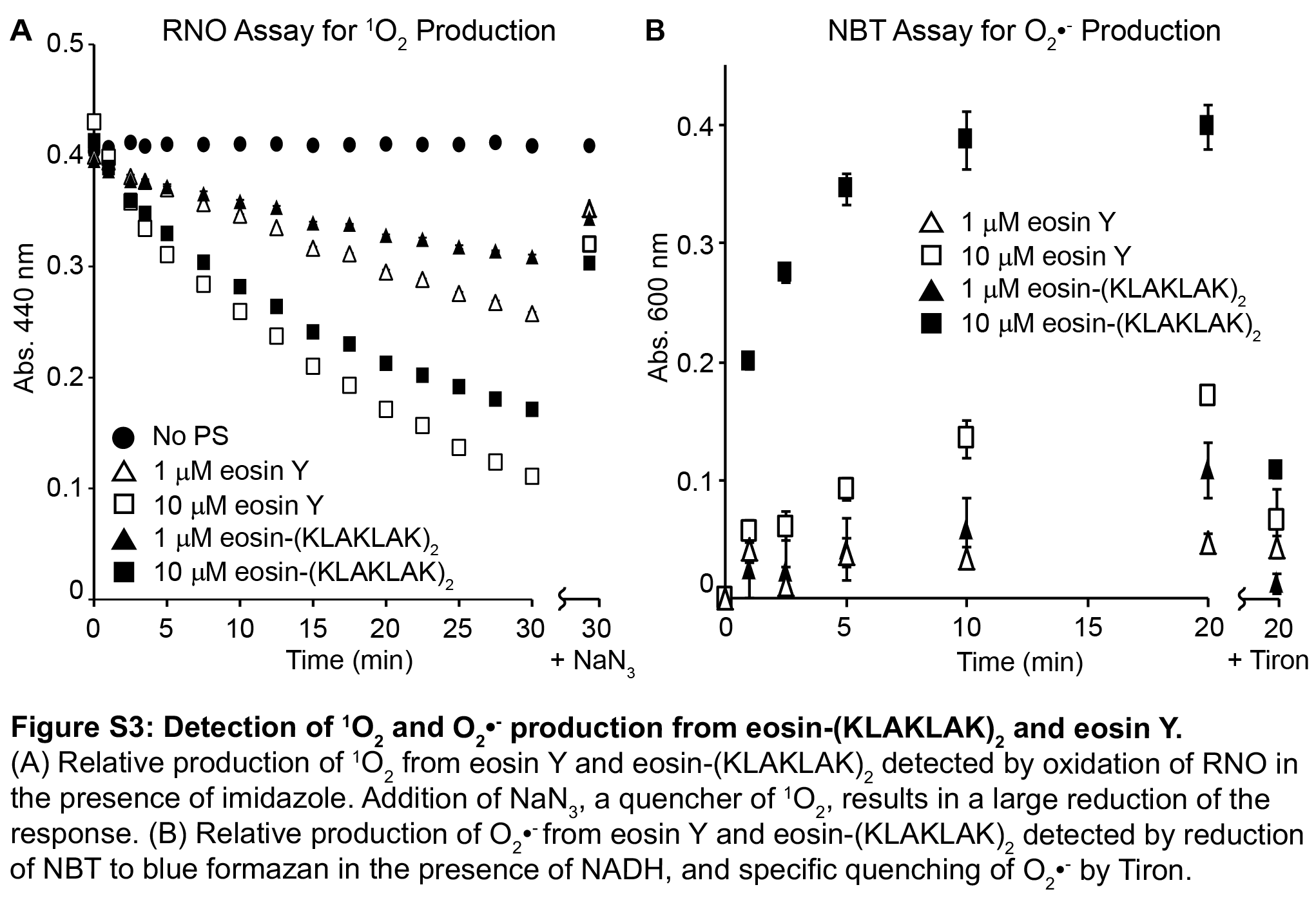

Supplement: Figure S3 — Detection of 1O2 and O2• − production from eosin-(KLAKLAK)2 and eosin Y. (A) Relative production of 1O2 from eosin Y and eosin-(KLAKLAK)2 detected by oxidation of RNO in the presence of imidazole. Addition of NaN3, a quencher of 1O2, results in a large reduction of the response. (B) Relative production of O2 •− from eosin Y and eosin-(KLAKLAK)2 detected by reduction of NBT to blue formazan in the presence of NADH, and specific quenching of O2 •− by Tiron. (TIF) [file pone.0091220.s003.tif]

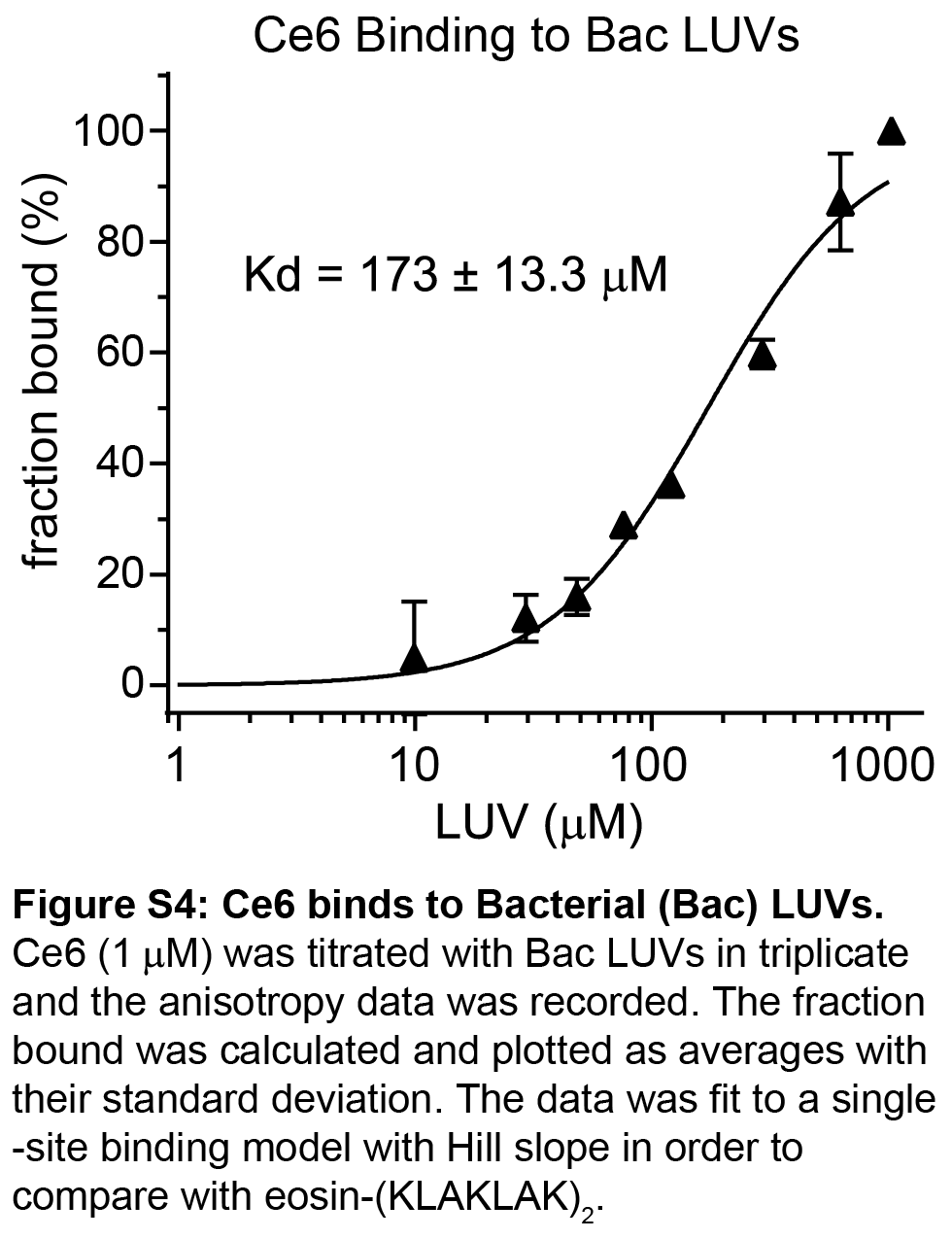

Supplement: Figure S4 — Ce6 binds to Bacterial (Bac) LUVs. Ce6 (1 µM) was titrated with Bac LUVs in triplicate and the anisotropy data was recorded. The fraction bound was calculated and plotted as averages with their standard deviation. The data was fit to a single site binding model with Hill slope in order to compare with eosin-(KLAKLAK)2. (TIF) [file pone.0091220.s004.tif]
